# Automated segmentation of magnetic resonance bone marrow signal: a feasibility study

**DOI:** 10.1007/s00247-021-05270-x

**Published:** 2022-02-02

**Authors:** Elisabeth von Brandis, Håvard B. Jenssen, Derk F. M. Avenarius, Atle Bjørnerud, Berit Flatø, Anders H. Tomterstad, Vibke Lilleby, Karen Rosendahl, Tomas Sakinis, Pia K. K. Zadig, Lil-Sofie Ording Müller

**Affiliations:** 1 Division of Radiology and Nuclear Medicine, Oslo University Hospital, Sognsvannsveien 20, 0372 Oslo, Norway; 2 Department of Medicine, Institute of Clinical Medicine, University of Oslo, Oslo, Norway; 3 Department of Radiology, University Hospital of North-Norway, Tromsø, Norway; 4 Department of Health Sciences, University of Tromsø, Tromsø, Norway; 5 Department of Physics, University of Oslo, Oslo, Norway; 6 Department of Rheumatology, Oslo University Hospital, Oslo, Norway

**Keywords:** Adolescents, Artificial intelligence, Bone marrow, Children, Convolutional neural network, Magnetic resonance imaging, Segmentation

## Abstract

**Background:**

Manual assessment of bone marrow signal is time-consuming and requires meticulous standardisation to secure adequate precision of findings.

**Objective:**

We examined the feasibility of using deep learning for automated segmentation of bone marrow signal in children and adolescents.

**Materials and methods:**

We selected knee images from 95 whole-body MRI examinations of healthy individuals and of children with chronic non-bacterial osteomyelitis, ages 6–18 years, in a longitudinal prospective multi-centre study cohort. Bone marrow signal on T2-weighted Dixon water-only images was divided into three color-coded intensity-levels: 1 = slightly increased; 2 = mildly increased; 3 = moderately to highly increased, up to fluid-like signal. We trained a convolutional neural network on 85 examinations to perform bone marrow segmentation. Four readers manually segmented a test set of 10 examinations and calculated ground truth using simultaneous truth and performance level estimation (STAPLE). We evaluated model and rater performance through Dice similarity coefficient and in consensus.

**Results:**

Consensus score of model performance showed acceptable results for all but one examination. Model performance and reader agreement had highest scores for level-1 signal (median Dice 0.68) and lowest scores for level-3 signal (median Dice 0.40), particularly in examinations where this signal was sparse.

**Conclusion:**

It is feasible to develop a deep-learning-based model for automated segmentation of bone marrow signal in children and adolescents. Our model performed poorest for the highest signal intensity in examinations where this signal was sparse. Further improvement requires training on larger and more balanced datasets and validation against ground truth, which should be established by radiologists from several institutions in consensus.

**Supplementary Information:**

The online version contains supplementary material available at 10.1007/s00247-021-05270-x.

## Introduction

Bone marrow oedema is an important feature on MRI in musculoskeletal disorders in children and adolescents for detecting disease and in scoring systems for monitoring disease activity [1–6]. Bone marrow oedema is defined as increased signal intensity on T2-weighted (T2-W) images with fat suppression with corresponding low signal on T1-weighted (T1-W) sequences [7] and is often diffuse and ill-defined. The signal is nonspecific and simply represents increased water content [8, 9] as compared to the surrounding tissue. In children and adolescents, the normal skeletal maturation processes can influence the MRI signal in a similar way as pathology. Consequently, pathological and normal signal intensities and patterns can overlap [10–12], particularly at the knee [13].

**Fig. 1:**
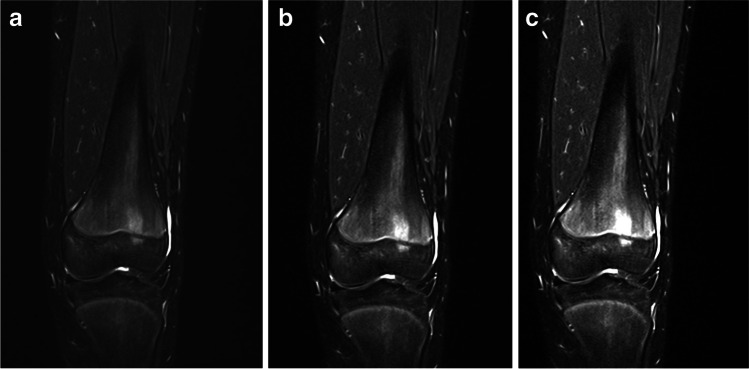
a–c MRI, coronal T2-W Dixon water-only of the knee in a healthy and asymptomatic 14-year-old girl. The perceived intensity level of the periphyseal bone marrow hyperintensity in the distal femur varies considerably with different window (*W*) and level (*C*) settings: (**a**) C192/W501, (**b**) C122/W271 and (**c**) C96/W198

Manual assessment of bone marrow signal is time-consuming and has been shown to be difficult because the perception of signal intensity inevitably changes with the surrounding background intensities [14–16] and there are challenges in standardising the signal intensity scale on MRI [17] (Fig. 1). In addition, perception of intensity values and image patterns is heavily influenced by subjective factors, e.g., the reader’s individual experience and expectations [18]. Acceptable intra- and interobserver variation for assessing bone marrow signal and extension require a meticulous calibration process [3, 5].

Artificial intelligence (AI) algorithms have, over the last few years, shown ground-breaking success in tackling tedious and difficult evaluation tasks. In deep learning, networks of simple interconnected units are used to recognise patterns and learn complex data representations [19] and are “often robust against undesired variation, such as the inter-reader variability” [20]. The most common type of deep neural network is a deep convolutional neural network (CNN). In radiology, the three main applications of deep CNNs are detection, classification, and segmentation. In detection and classification tasks, objects are detected in an image and classified, e.g., as benign or malignant, whereas in a segmentation task, an image is divided into different regions to separate distinct parts or objects, often as a pre-processing step to extract and classify features [19].

Automated models based on deep learning algorithms have recently been proposed as a problem-solving tool for detecting and classifying bone marrow pathology, both in adults and children [21, 22]. These studies presupposed a definition of pathological bone marrow signal on MRI, although to date, no such objective definition exists. A recent paper by Zhao et al. [5] suggested that AI models might increase both precision and accuracy of bone marrow imaging. However, training an AI model to distinguish between normal and pathological bone marrow signal requires consistent data input of both normal and pathological signal intensities and patterns, which in turn depend on consistent reading of the MR images.

A necessary pre-processing step in the development of a machine-learning model for detecting bone marrow pathology would be to develop a model for segmentation of various bone marrow signals, ranging from normal to abnormal in both healthy and diseased individuals, independent of the clinical context. The next step would be to include clinical data and train a model to identify and delineate pathological marrow signal and patterns.

In children and adolescents, the skeletal anatomy and bone marrow signal vary with age, and some anatomical structures other than bone marrow return high signal on MRI, e.g., the physis. The aim of our study was to explore the feasibility of an automated method for segmentation of bone marrow hyperintensities in the growing skeleton, including both healthy individuals and children with chronic non-bacterial osteomyelitis. Further, we wanted to pinpoint areas of improvement for developing a universally accepted model for clinical and research applications. To our knowledge this is the first study to address the feasibility of automated bone marrow signal segmentation on MRI in children and adolescents. We hypothesised that by using a two-dimensional (2-D) CNN it is possible to develop an automated model that can recognise different levels of bone marrow signal on MRI in the paediatric age group, where anatomy varies with age. Further, the model should avoid structures with high signal other than bone marrow.

## Materials and methods

The project was approved by the regional ethics committee (no 2016/1696). We obtained written informed consent from all the participants or their caregivers for participation in the study and publication of the data.

### Study population

This study is part of a longitudinal prospective multi-centre project to establish an MRI-based scoring system for the paediatric skeleton on whole-body MRI to describe variations in bone marrow signal in healthy children and adolescents. During the period from March 2018 to March 2020, 196 healthy children and adolescents ages 6–18 years residing in Tromsø and Oslo underwent a whole-body MRI for research purposes. Thirty children with chronic non-bacterial osteomyelitis who were examined with the same whole-body MRI protocol were also invited to participate in the study.

### Dataset

We selected 95 whole-body MRI examinations (68 from Oslo, 27 from Tromsø). Images from both healthy individuals (67) and children with chronic non-bacterial osteomyelitis (28), ages 6–18 years, were included. The selection was carefully done to ensure a balanced data heterogeneity of bone marrow signal and an even age distribution throughout the cohort to avoid bias in the training process. Images with artifacts were excluded.

We used 85 examinations for training and validation of the model and manually selected a test set of 10 examinations with representative age distribution and bone marrow signal for evaluating model performance and inter-rater variance [23]. Each examination had an average of 15.3 slices for segmentation, for a total of 1,318 slices for training and 153 for testing. In machine learning, the division of training dataset and validation/test dataset is traditionally done by selecting the largest possible amount of data for training, typically 80%, and 10% for the validation dataset during the training process and 10% for the final test set [24]. MRI protocol examinations were performed at two institutions on 1.5-tesla (T) MRI scanners (Magnetom Aera by Siemens Healthcare, Oslo, Norway; and Ingenia by Philips Healthcare, Tromsø, Norway). The imaging protocol consisted of a coronal scan from skull to feet in 3–5 steps with the following sequences: T1-W, T2-W and diffusion-weighted (DWI) sequences, performed during free breathing. Total scan time was approximately 30–45 min. All participants either watched a movie or listened to music during the examination. Sedation was not used.

For the current study, we selected the T2-W Dixon water-only and fat-only images of the knee region with the following scan parameters: repetition time/echo time [TR/TE] = 4,700/109 ms with voxel size 0.9 × 0.9 × 3.5 mm.

### Segmentation and training

**Fig. 2:**
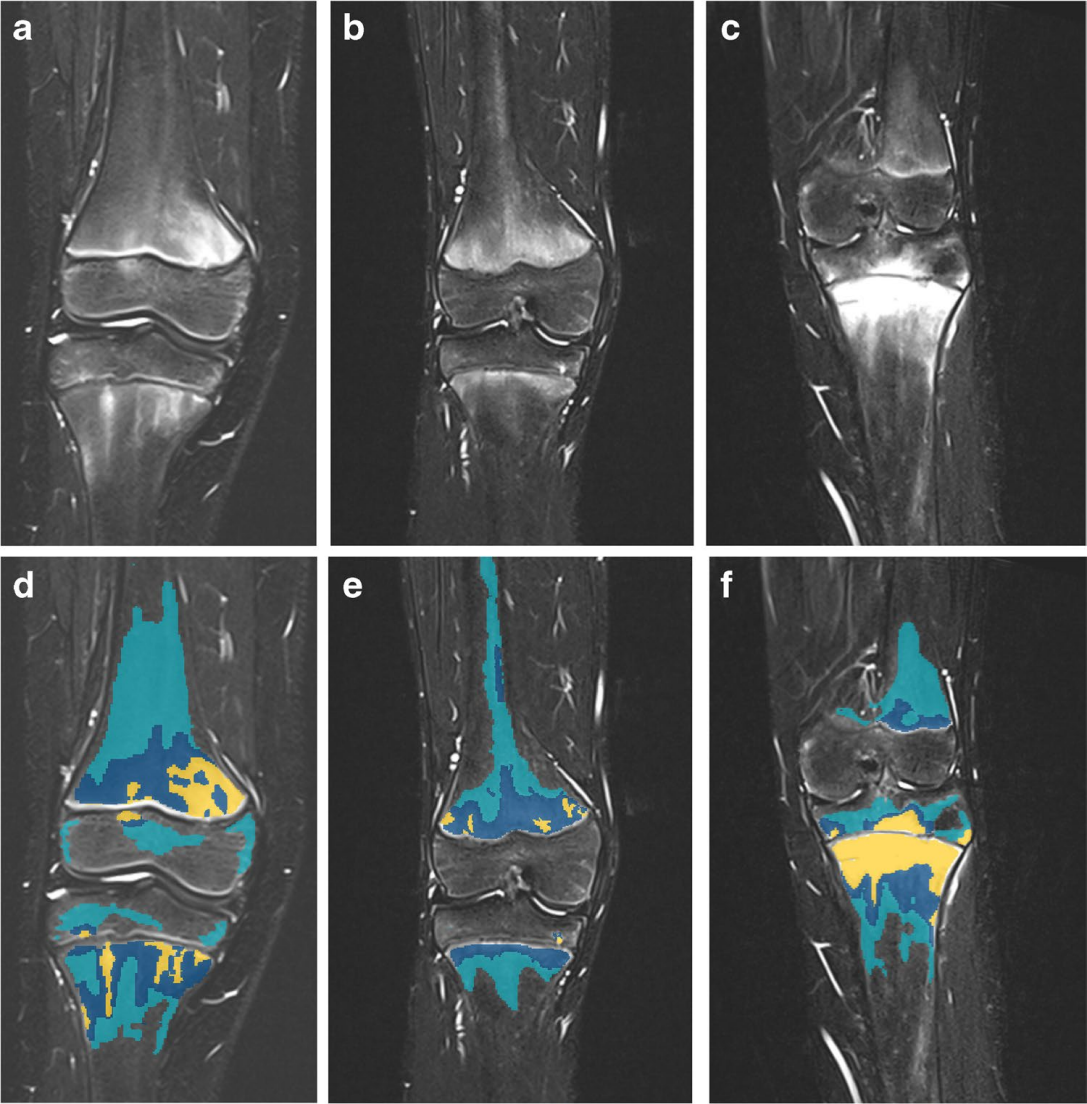
Bone marrow signal was divided into three intensity levels: 1 = slightly increased with diffuse distribution; 2 = focal and mildly increased; and 3 = focal and moderately to highly increased, up to fluid-like signal (turquoise = level 1, blue = level 2, yellow = level 3). These images illustrate the defined intensity levels on coronal T2-W Dixon water-only images of the knee (**a–c**) with corresponding segmentation masks (**d–f)** in a 12-year-old boy with chronic non-bacterial osteomyelitis and knee symptoms (**a, d**), a 14-year-old healthy and asymptomatic boy (**b, e**) and a 15-year-old girl with chronic non-bacterial osteomyelitis and knee symptoms (**c, f**)

**Fig. 3:**
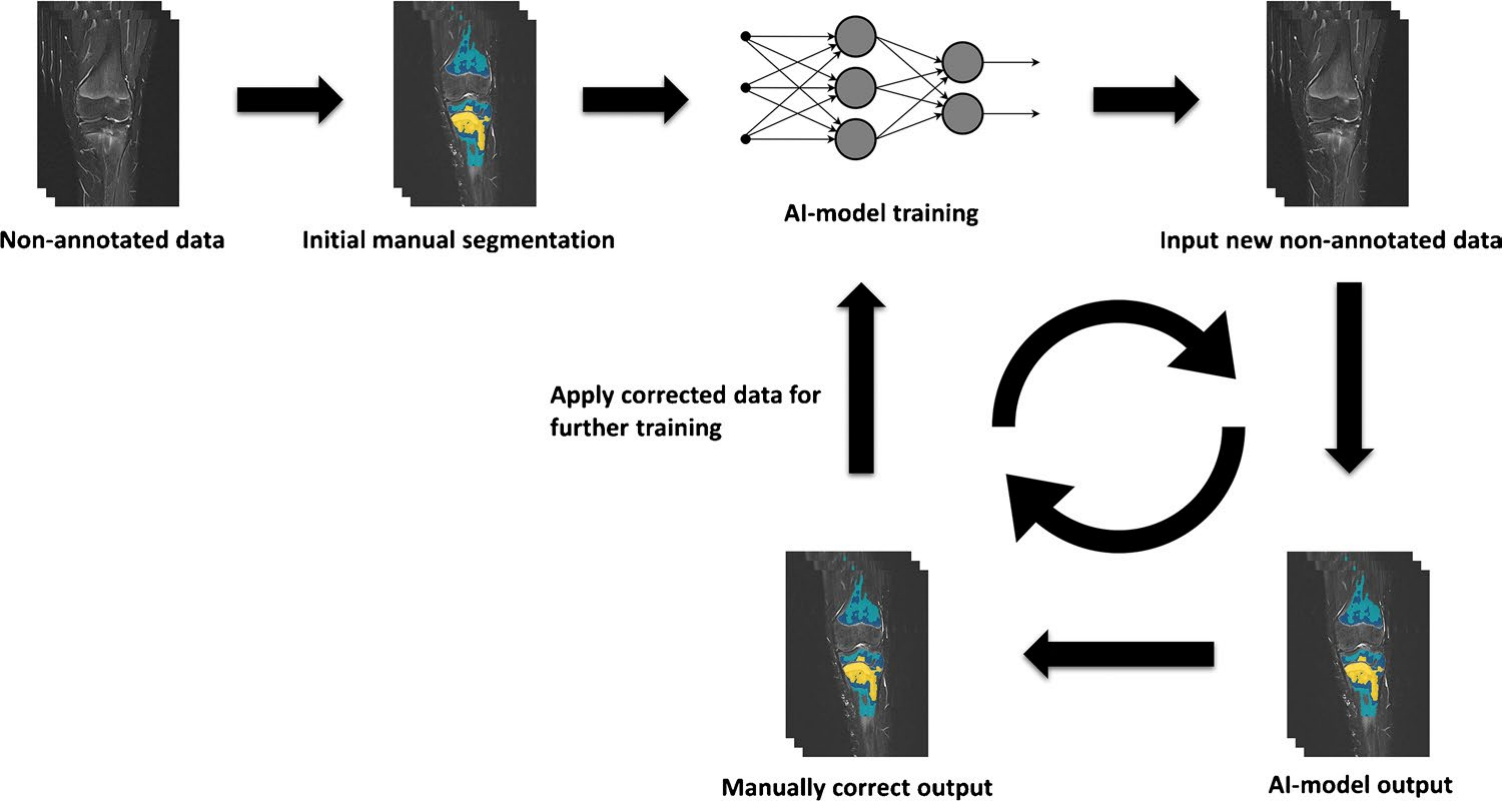
Simplified illustration of the iterative segmentation process. The model, which is initially trained on small amounts of data, contributes output that is then manually corrected to further produce training data. This process cuts down on data preparation time and helps to identify areas the preliminary model is struggling with, which allows for focused adjustment of hyperparameters and network architecture to resolve the largest systematic errors. *AI* artificial intelligence

All images were converted into Neuroimaging Informatics Technology Initiative (NIfTI) files prior to segmentation, a simpler and more standardised file format than the Digital Imaging and Communications in Medicine (DICOM) format. NIfTI files are fully anonymised and commonly used in AI training. The readers were blinded to clinical information, age and institution during the segmentation process. We used T2-W Dixon water-only sequences for segmentation of bone marrow signal at the metaphyses and epiphyses of the distal femur and the proximal tibia of both knees. We made efforts to standardise the reading conditions prior to segmentation with respect to room lighting and by window levelling so that air appeared black with a clearly defined boundary to subcutaneous tissue. Bone marrow signal intensity was divided into three categories: level 1 = slightly increased with diffuse distribution; level 2 = focal and mildly increased; level 3 = focal and moderately to highly increased, up to fluid-like signal as compared to the lowest signal of the fatty marrow. Two radiologists (E.vB. and L.-S.O.M., both with 15 years of experience in paediatric musculoskeletal radiology) defined the intensity range for each intensity level in consensus and elaborated a reference atlas. They applied masks with colour-coding, representing the three grades of signal intensity, to each slice of both knees (Fig. 2) using the web-based segmentation programme MedSeg [25], avoiding physeal lines and obvious vessels. The model was trained by one radiologist (E.vB.) using U-net-aided iterative segmentation. U-net refers to the specific subtype of the deep learning model used [26]. Iterative segmentation is a process where a small part of the dataset is initially manually segmented. This small dataset is then used to train a rudimentary segmentation model, which is further used on new, unsegmented data. The rudimentary model is used to accelerate the manual preparation process by allowing error. A simplified illustration of the iterative segmentation process is provided in Fig. 3. A complete version of the training process can be obtained from Supplementary Online Material 1. Using MedSeg, the same reader marked the anatomical region of interest. The metaphysis was defined by a square over the growth plate of the affected bone, each side with a length equal to the maximum width of the epiphysis [27].

### Evaluation

The radiologists involved in the initial calibration and training (readers 1 and 2), an MRI radiographer from the same institution (reader 3, A.H.T., with limited experience in clinical grading of bone marrow signal) and a paediatric musculoskeletal radiologist from a different institution (reader 4, K.R., with 30 years of experience) manually segmented the bone marrow signal on each image slice in the 10 examinations from the test set. In total each reader independently segmented 153 image slices. For calibration prior to the evaluation task, the readers were provided with a reference atlas consisting of masks from 10 knee MRIs that were not included in the test set. The atlas comprised the whole range of bone marrow signal intensities. In addition, reader 3 (A.H.T.) underwent a thorough calibration session with reader 1 (E.vB.) prior to the evaluation exercise. They established ground truth by using the simultaneous truth and performance level estimation (STAPLE) algorithm [28] based on the segmentation masks from all four readers.

Model performance and differences between readers were evaluated through the Dice similarity coefficient (“Evaluation metrics” section). In addition, masks derived from the ground truth and model for the whole training set were randomised and evaluated in consensus by readers 1, 2 and 3. The readers were blinded to age, institution and whether the segmentation was performed by the AI model or represented ground truth. To ascertain the latter, reader 4 (K.R.) assessed the test set to look for and potentially remove markings outside the bone marrow prior to the reading. Readers scored the three different signal intensities separately for each segmentation mask in the test set. In addition, they gave an “overall impression” score to each mask. The rating was performed using a visual analogue scale (VAS), i.e. a two-sided ruler with the minimum and maximum scores on one side of the ruler, and a 10-cm-long line with centimetre marks on the back for improved discrimination of the scores [29, 30]. A sliding marker shows the same spot on the 10-cm line on both sides of the ruler (illustrated in [30]). For the fixed-point scale, 1 = perfect segmentation; 2 = minor corrections needed, less likely to have clinical impact; 3 = major corrections needed, most likely to have clinical impact; and 4 = mask rejected. For the continuous scale, values less than 5 were deemed acceptable (this corresponds to a score midway between points 2 and 3 on the fixed-point scale).

### Evaluation metrics

We used Dice similarity coefficient to measure the volume-based similarity between the segmentation masks. The more overlap of the masks, the larger the Dice coefficient. The value of the Dice coefficient is always between 0 and 1 [31]. We performed descriptive statistical analyses using Predictive Analytics Software (SPSS) version 27 (IBM, Armonk, NY).

## Results

### Dice similarity coefficient

Table 1 lists the median and mean Dice coefficients between estimated ground truth and the segmentations performed by the AI model and the four readers (range of values in brackets).

**Table 1:** Median and mean Dice similarity coefficient between ground truth and the segmentations performed by the artificial intelligence (AI) model and the four readers

		Reader 1	Reader 2	Reader 3	Reader 4	AI model
Level-1 signal (turquoise)	Median (range)	0.80 (0.69–0.90)	0.83 (0.70–0.88)	0.73 (0.42–0.81)	0.75 (0.57–0.84)	0.68 (0.60–0.74)
	Mean	0.81	0.8	0.67	0.72	0.68
Level-2 signal (blue)	Median (range)	0.72 (0.35–0.87)	0.67 (0.23–0.84)	0.55 (0.29–0.78)	0.17 (0.01–0.53)	0.47 (0.25–0.62)
	Mean	0.7	0.64	0.55	0.17	0.45
Level-3 signal (yellow)	Median (range)	0.64 (0.20–0.87)	0.67 (0.52–0.93)	0.59 (0.44–0.75)	0.00 (0.00–0.89)	0.40 (0.00–0.71)
	Mean	0.6	0.69	0.59	0.11	0.38
Combined levels 2 + 3 signal	Median (range)	0.79 (0.33–0.86)	0.71 (0.19–0.89)	0.61 (0.38–0.78)	0.18 (0.01–0.73)	0.55 (0.24–0.74)
	Mean	0.7	0.69	0.6	0.21	0.5

**Fig. 4:**
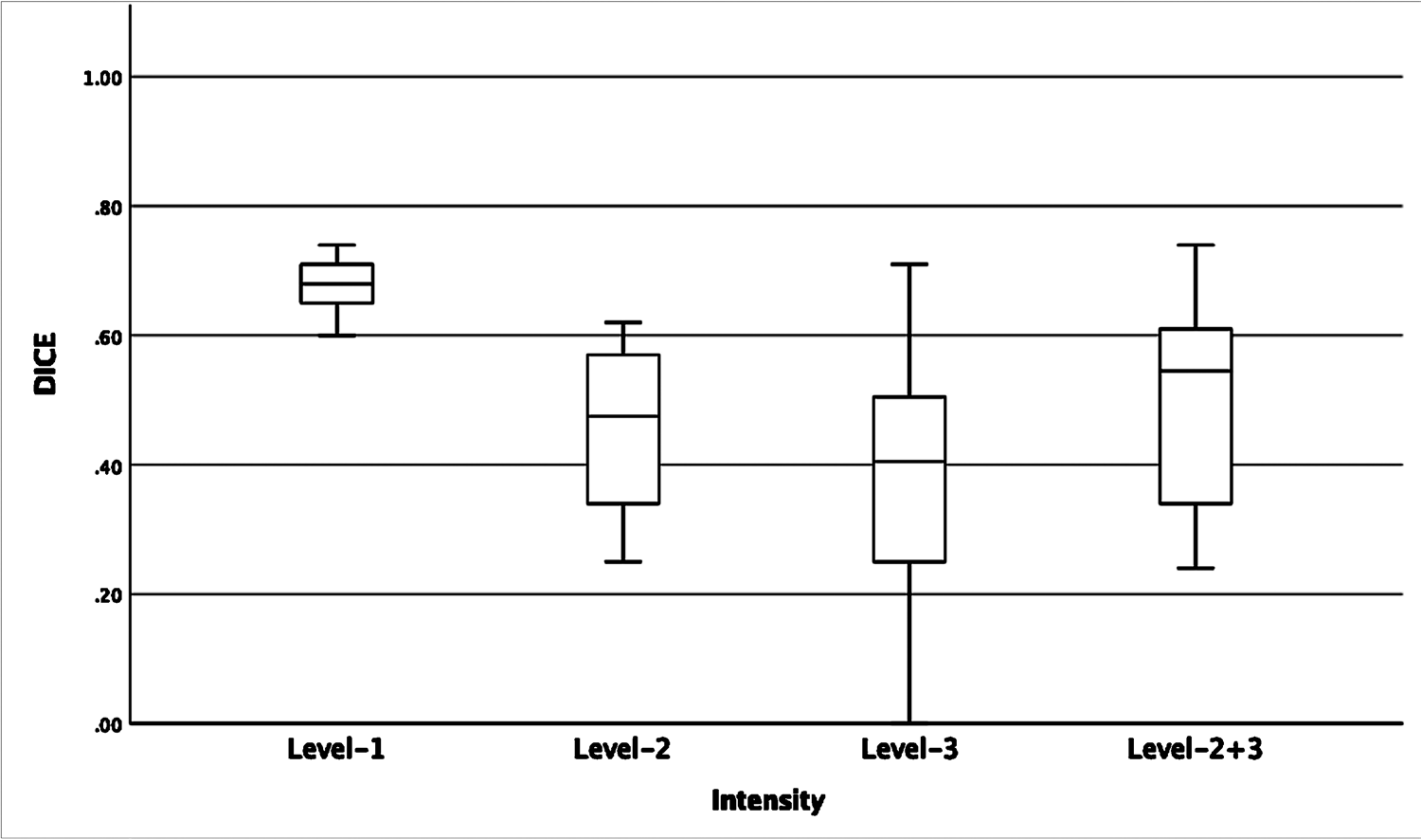
Boxplot illustrates the performance of the artificial intelligence (AI) model compared to ground truth for the different signal intensities expressed by the Dice similarity coefficient

The AI model’s highest Dice coefficient was for level-1 signal, with median Dice 0.68 (0.60–0.74), followed by level-2 signal, with median Dice 0.47 (0.25–0.62). The model scored lowest for the highest intensity level, with median Dice of 0.40 (0–0.71). The Dice coefficient of 0 for the highest intensity level was obtained in one examination only. This was in a healthy subject with no level-3 signal present in the segmentation masks performed by the AI model and reader 4, whereas a few small spots of high signal were defined to be present according to the ground truth and readers 1–3. A boxplot illustrating the performance of the AI model compared to ground truth for the different signal intensities is presented in Fig. 4.

Difference between readers was smallest for level 1 signal, with median Dice for the four raters compared to ground truth between 0.68 and 0.83. For the two other intensity levels, the differences were greater: median Dice ranged 0.17–0.72 for level-2 signal and 0–0.67 for level-3 signal. One rater consequently achieved a Dice coefficient of 0 for level-3 signal in all but one examination and had by far the lowest mean Dice for both level-2 (0.17) and level-3 (0.11) signals. However, for level-1 signal, the mean Dice of 0.72 was at a similar level as that of the other raters (0.67–0.81).

In general, the poorest performance of the model and the maximum difference between readers were observed for level-3 signal in examinations where this intensity level was sparse, both in healthy and sick individuals. In examinations where level-3 signal was more pronounced, the Dice coefficient increased correspondingly. The median Dice for the combination of intensity levels 2 + 3 was consequently higher for all four readers and the model, as compared to the separate evaluation of the two intensity levels.

### Consensus evaluation

The model did not draw any markings outside the bone marrow. Table 2 lists the consensus scores with means and standard deviations (SD) for each intensity level and for the overall impression of the segmentation masks representing ground truth and the segmentations performed by the model. The lower the score, the better the agreement.

**Table 2:** Consensus scores with means and standard deviations (SD) for each intensity level and for the overall impression of the segmentation masks representing ground truth (GT) and the segmentations performed by the artificial intelligence (AI) model, and for the differences between the two scores (Diff_AI-GT_)

Mask number	Number of slices with bone marrow signal	Age	Level-1 signal (turquoise)^a^			Level-2 signal (blue)^b^			Level-3 signal (yellow)^c^			Overall impression		
			GT	AI	Diff _AI-GT_	GT	AI	Diff _AI-GT_	GT	AI	Diff _AI-GT_	GT	AI	Diff _AI-GT_
1	16	14.0	3.4	3.4	0.0	3.2	3.4	0.2	3.4	5.0	1.6	3.4	5.0	1.6
2	16	13.9	1.5	5.0	3.5	2.4	5.0	2.6	1.5	6.8	5.3	2.0	6.0	4.0
3	15	10.2	3.4	3.4	0.0	3.5	3.4	–0.1	3.0	5.0	2.0	3.2	4.2	1.0
4	15	9.6	2.0	1.8	–0.2	3.0	1.8	–1.2	3.4	4.0	0.6	3.2	3.5	0.3
5	16	9.5	4.2	1.8	–2.4	5.0	1.8	–3.2	0	1.8	1.8	3.0	1.8	–1.2
6	15	7.6	1.8	3.0	1.2	3.4	3.8	0.4	6.8	3.8	3.8	4.0	3.8	–0.2
7	14	14.3	1.5	1.0	–0.5	1.5	3.4	1.9	0.5	3.8	3.8	1.0	3.5	2.5
8	16	7.8	1.0	0.5	–0.5	2.5	2.0	–0.5	0.5	0.5	0.5	2.0	1.0	–1.0
9	15	11.0	3.2	1.8	–1.4	3.4	0.5	–2.9	1.3	3.8	3.8	1.8	3.0	1.2
10	15	9.6	2.0	1.8	–0.2	2.0	2.0	0.0	0	0	0.0	1.0	1.5	0.5
Mean	15.30	10.8	2.40	2.35	–0.50	2.99	2.71	–0.28	2.04	3.45	1.41	2.46	3.33	0.87
SD	0.67	2.5	1.06	1.34	1.56	0.97	1.31	1.83	2.14	2.11	2.22	1.04	1.57	1.58

*SD* standard deviation

^a^Intensity level 1 = slightly increased with diffuse distribution

^b^Intensity level 2 = focal and mildly increased signal

^c^Intensity level 3 = focal and moderately to highly increased, up to fluid-like signal

The mean scores for both model and ground truth were all within the values defined as acceptable, both with respect to each intensity level and the overall impression. The overall impression of the model (mean 3.33 ± 1.57) was slightly poorer than that of the ground truth (mean 2.46 ± 1.04).

The results for the intensity level 1 (turquoise) were similar between model (mean 2.35 ± 1.34) and ground truth (mean 2.40 ± 1.06), whereas for the intensity level 2 (blue) the model achieved the best mean score (2.71 ± 1.31 vs. ground truth 2.99 ± 0.97). The model had the poorest performance on the intensity level 3 (yellow), with a tendency to underestimate particularly punctate and linear hyperintensities (mean score 3.45 ± 2.11). In one examination (mask number 1 in Table 2) the model underestimated level-3 signal on some slices, whereas it overestimated this intensity level on other slices. In one examination (mask number 2 in Table 2), the model performed poorer than acceptable for all intensity levels.

The model consistently avoided the physeal lines and vessels. Signal in the medial part of the femoral metaphysis was missed by the model in one peripheral slice. The general impression was that most inaccuracies were found in the peripheral slices.

## Discussion

We have shown that this model enables segmentation of a wide spectrum of bone marrow signal in children and adolescents where anatomy varies with age, while avoiding high signal structures other than bone marrow, on images obtained at two institutions on 1.5-T MRI machines from two vendors.

### Dice analyses of model performance

The Dice coefficient for the model varied substantially for each intensity level, with the highest mean value for level-1 signal and the lowest for level-3 signal. In total, the highest Dice value achieved by the model was 0.74. In bone marrow segmentation there is a gradual transition between the elements we intend to segment, unlike organ segmentation, where there is a more absolute delineation of structures. In addition, it is difficult to standardise window levelling for MRI reading [17], which in our case yielded very different impressions of the bone marrow signal and might alter decision-making when it comes to defining levels of signal (Fig. 1) and ultimately masks. We therefore argue that an AI model for automated bone marrow segmentation on MRI, with ground truth based on segmentations performed by several independent readers, might never achieve Dice coefficients close to 1 because this would entail a complete agreement among all readers behind the ground truth as well as complete agreement between ground truth and the annotator of the training data.

**Fig. 5:**
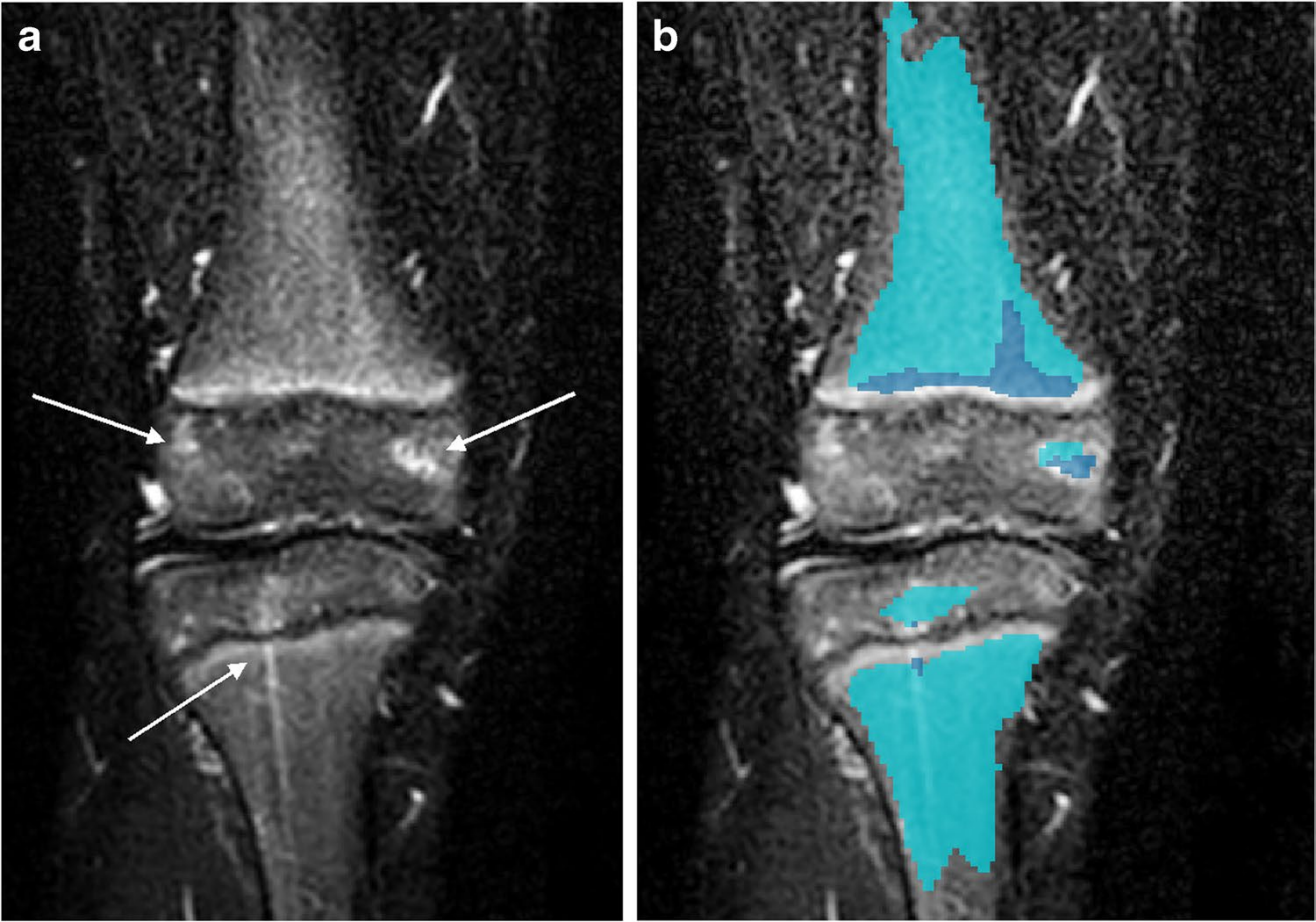
**a, b** MRI, coronal T2-W Dixon water-only of the knee in an 8-year-old healthy and asymptomatic girl. Image (**b**) includes the segmentation mask performed by the model. The small foci of level-3 signal shown with *arrows* in image (**a**) are either missed (no color coding in image **b**) or incorrectly labelled with a lower intensity level by the model (coded with either blue or turquoise in image **b**)

In two knees in the test set, no signal was deemed level 3, either by the four readers or the model. One could argue that this expresses full agreement; however, the Dice coefficient cannot be calculated when there are no data. In one knee, the model did not recognise any signal with intensity level 3, whereas the segmentation mask representing ground truth contained a few minor yellow spots (Fig. 5). The resultant Dice coefficient of 0 considerably influenced the mean Dice.

We observed low Dice values for level-3 signal in all knees where the amount of level-3 signal was relatively sparse. In these datasets, only small inaccuracies in segmentation have a major effect on the Dice coefficient [32]. Variations in segmentation of signal intensities with a more diffuse and widespread distribution would have less impact on the Dice coefficient. This might explain the better performance of the model and all four readers for segmentation of intensity level 1, for the combination of intensity levels 2 and 3, and for intensity level 3 in examinations where this signal was more pronounced.

### Reader variability

It was beyond the scope of our study to determine the most accurate reader in terms of clinical validity. The Dice coefficients varied considerably among the readers, in particular between readers 1–3 and reader 4. Reader 3 has limited clinical experience with image interpretation. Interestingly, agreement between this reader and the experienced readers 1 and 2 was substantially better than the agreement between readers 1 and 2, and the experienced reader 4. Reader 4 had the most experience with paediatric musculoskeletal imaging, but readers 1–3 were from the same institution and readings were performed under similar conditions in terms of workstation, room lighting and window levelling. In addition, the first three readers underwent a thorough calibration process prior to segmentation of the test set. This illustrates that the establishment of consistent definitions of different bone marrow signal intensities to a lesser degree depends on the individual radiologist's clinical experience with bone marrow imaging than thorough calibration sessions.

### Consensus evaluation of model performance

We found the consensus scoring particularly useful as a complementary evaluation tool to the Dice coefficients in this study. Consensus reading implies a common effort targeted toward agreement. In some settings, particularly where efforts are made to establish definitions, consensus reading can be the most applicable method for image interpretation [33]. The consensus reading allowed for more specific description of areas where the AI model was suboptimal and, as opposed to the Dice similarity coefficient, the consensus reading enabled differentiation of clinically significant from insignificant errors and identification of focus areas for further training of the model. Results from consensus scoring showed that the model performed well except for one examination, where the model missed punctate level-3 signal foci (Fig. 5). We think the reason for this is that punctate and linear bone marrow hyperintensities other than obvious vessels were relatively sparse in our training dataset.

We observed most inaccuracies on peripheral slices, probably because the transition between bone marrow and soft tissue is less conspicuous in the periphery on coronal T2-W Dixon water-only images and there is partial volume effect in this area. Overall, the results from this subjective evaluation of the model reflect the results from the Dice analyses, with respect to the mean scores for the three signal intensities. Interestingly, the Dice coefficient did not correspond with the consensus scores for every individual knee. This observation confirms that the Dice coefficient does not always reflect the clinical relevance of an inaccuracy.

### Training dataset

To ensure sufficient data for all intensity levels, we trained this model on an anatomical site with a high incidence of bone marrow signal hyperintensities in both healthy and sick children and adolescents. Patchy or flame-shaped patterns of residual red bone marrow are typically seen in this location, especially between the ages of 11 years and 15 years [34] and can easily be mistaken for inflammatory changes [35]. An automated segmentation model for bone marrow hyperintensities could therefore prove particularly useful for the knee, but we think this technique could also be used to train models for other anatomical sites. The highest signal-intensity level is most likely to be interpreted as pathological. This signal intensity could be further divided into two levels to improve clinical validity; however, level 3 signal was far less frequent and less extensive when present, compared to the two other intensities. This poses a challenge for the training process. To train a more robust model, more images with high signal intensity and pathological bone structure should be included in the training dataset.

### Segmentation process

Following the initial calibration, one radiologist (E.vB.) performed the segmentation of the training dataset to ensure consistency throughout the training process. The reader reported that it was difficult to determine the level of signal intensity because there is no clear cut-off for each level and there is sometimes even a range of signal intensity within each level, particularly for level 3, where the signal could range up to fluid-like (Fig. 2). It was also difficult to standardise window-level settings, which again reflects that consistent scoring of signal intensity on MRI is challenging (Fig. 1). Time spent to correct masks is sometimes used as a measure to evaluate a segmentation model [36]. We did not find this useful in our study because the amount and intensity of bone marrow signal varied considerably among the examinations, which itself influences the time it takes both for segmentation from scratch and correction of masks.

### Limitations

The study has some limitations that could be addressed in future research. First, definitions of bone marrow intensities were from one single institution, hence a multicentre consensus-based definition might differ from ours. However, the scope of this study was only to explore the feasibility of automated bone marrow segmentation based on predefined signal intensities and to identify the challenges one might face when developing a model for bone marrow segmentation. The aim was not to develop a universally applicable segmentation model because this would require a wider international collaboration.

Second, in the paediatric age group there is a wide spectrum of skeletal and bone marrow appearances, and we cannot ensure that testing the model on our test set reflects all potential errors. Our test set of 10 examinations (including a total of 153 coronal images of bone marrow from two knees) was carefully selected to ensure the most optimal data heterogeneity of bone marrow signal to avoid bias. Bone marrow signal is more likely to be sparse in the oldest age group [34]; hence, our test set did not include individuals older than 15 years. One could argue that this does not provide a representative age distribution for age-dependent anatomy; however, we included the ages where the anatomical changes related to growth and maturation are most pronounced. Our test set was not a random selection because assessment of the differences in model and reader agreement with age was not within the scope of this paper.

The lack of an objective reference for the bone marrow signal intensity is another limitation of this study. This is indeed the limitation for subjective bone marrow interpretation in all settings. We acknowledge that the segmentation process is hampered by subjectiveness and, consequently, that our model was trained on in part inconsistent data. However, when training on a large dataset, these inconsistencies are averaged by the model.

Further, the poorest performance of our model was seen with the highest — and most likely abnormal — signal, in examinations where this signal was sparse. However, not only the signal intensity, as such, but also the *pattern and extent* of signal distribution are important features for the interpretation of bone marrow signal. In children with inflammatory changes, level-3 intensity rarely occurred without the presence of surrounding level-2 signal and often level-1 signal also. Depending on the clinical setting, widespread or certain distribution of level-2 intensities could be more likely to represent pathology than small focal spots of level-3 signal intensities. In our cohort, the highest intensity level was sparse in both healthy and sick individuals. We believe that further training, which would require more training data containing level-3 signal intensity, could improve this shortcoming.

Consensus reading implies a common effort targeted toward agreement, but this is hampered by subjective bias and therefore discussed as a limitation in most radiologic studies. However, in some settings, particularly where efforts are made to establish definitions, consensus reading is the most applicable method for image interpretation [33]. During development of the segmentation model, we considered consensus reading to be a useful supplementary method to the more objective Dice coefficient for evaluating the model. Consensus reading provides the opportunity to describe model performance in more detail, e.g., to discuss clinically significant versus insignificant errors, and identify areas for further improvement of the model.

Finally, our data were trained and tested on MRI examinations from two institutions where the images were obtained with similar MRI parameters, hence we did not test robustness for other vendors or protocols.

### Strengths

The strengths of this study are the inclusion of both healthy subjects and subjects with chronic non-bacterial osteomyelitis. We included datasets from two institutions with two MRI vendors. In addition to the objective standard evaluation methods of AI models, we performed a subjective, more clinically directed evaluation. This is the first study of its kind and might serve as a valuable first step for developing an international ground truth database to serve for validation purposes applicable to research and clinical practice.

### Future perspectives

To our knowledge, this is the first study addressing the feasibility of using a deep-learning-based model for automated segmentation of both normal and pathological bone marrow signal on MRI in the paediatric population. One potential approach for future improvement of the segmentation model would be to collect sufficient training data for all signal intensities and patterns and make this publicly available for an international segmentation challenge.

To develop a robust and universally accepted model for bone marrow segmentation, definition and training performed in consensus by a larger international group is crucial, as also highlighted by Zhao et al. [5]. In this study we tested a fairly standard 2-D U-net model. Newer CNN architectures might perform better in the future.

## Conclusion

We have shown that it is feasible to develop an automated method for segmentation of bone marrow signal in children and adolescents using a 2-D CNN. We found that the highest intensity level in examinations where this signal was sparse had the poorest performance. Improvement of the model requires training on larger and more balanced datasets. Further development of the model and validation of the segmented bone marrow intensities should be performed by radiologists from several institutions in consensus to achieve the most robust results.

## Supplementary Information

Below is the link to the electronic supplementary material.

Supplementary file1 (DOCX 14 kb)

## References

[1] Hemke R, Tzaribachev N, Nusman CM (2017). Magnetic resonance imaging (MRI) of the knee as an outcome measure in juvenile idiopathic arthritis: an OMERACT reliability study on MRI scales. J Rheumatol.

[2] Herregods N, Dehoorne J, Van den Bosch F (2017). ASAS definition for sacroiliitis on MRI in SpA: applicable to children?. Pediatr Rheumatol Online J.

[3] Tanturri de Horatio L, Damasio MB, Barbuti D (2012). MRI assessment of bone marrow in children with juvenile idiopathic arthritis: intra- and inter-observer variability. Pediatr Radiol.

[4] Weiss PF, Maksymowych WP, Lambert RG (2018). Feasibility and reliability of the Spondyloarthritis Research Consortium of Canada sacroiliac joint inflammation score in children. Arthritis Res Ther.

[5] Zhao Y, Sato TS, Nielsen SM (2019). Development of CROMRIS (chronic nonbacterial osteomyelitis MRI scoring) tool and evaluation of its interrater reliability. J Rheumatol.

[6] Panwar J, Tse SML, Lim L (2019). Spondyloarthritis Research Consortium of Canada scoring system for sacroiliitis in juvenile spondyloarthritis/enthesitis-related arthritis: a reliability, validity, and responsiveness study. J Rheumatol.

[7] Ostergaard M, Peterfy C, Conaghan P (2003). OMERACT rheumatoid arthritis magnetic resonance imaging studies. Core set of MRI acquisitions, joint pathology definitions, and the OMERACT RA-MRI scoring system. J Rheumatol.

[8] Jimenez-Boj E, Nobauer-Huhmann I, Hanslik-Schnabel B (2007). Bone erosions and bone marrow edema as defined by magnetic resonance imaging reflect true bone marrow inflammation in rheumatoid arthritis. Arthritis Rheum.

[9] McQueen FM, Ostendorf B (2006). What is MRI bone oedema in rheumatoid arthritis and why does it matter?. Arthritis Res Ther.

[10] Avenarius DFM, Ording Muller LS, Rosendahl K (2017). Joint fluid, bone marrow edemalike changes, and ganglion cysts in the pediatric wrist: features that may mimic pathologic abnormalities — follow-up of a healthy cohort. AJR Am J Roentgenol.

[11] Ording Muller LS, Avenarius D, Damasio B (2011). The paediatric wrist revisited: redefining MR findings in healthy children. Ann Rheum Dis.

[12] Shabshin N, Schweitzer ME, Morrison WB (2006). High-signal T2 changes of the bone marrow of the foot and ankle in children: red marrow or traumatic changes?. Pediatr Radiol.

[13] Maraghelli D, Brandi ML, Matucci Cerinic M (2021). Edema-like marrow signal intensity: a narrative review with a pictorial essay. Skeletal Radiol.

[14] Diamon AL (1953). Foveal simultaneous brightness contrast as a function of inducing, and test-field luminances. J Exp Psychol.

[15] Leibowitz H, Mote FA, Thurlow WR (1953). Simultaneous contrast as a function of separation between test and inducing fields. J Exp Psychol.

[16] Sinha P, Crucilla S, Gandhi T (2020). Mechanisms underlying simultaneous brightness contrast: early and innate. Vision Res.

[17] Nyúl LG, Udupa JK (1999). On standardizing the MR image intensity scale. Magn Reson Med.

[18] Brady AP (2017). Error and discrepancy in radiology: inevitable or avoidable?. Insights Imaging.

[19] Mazurowski MA, Buda M, Saha A, Bashir MR (2019). Deep learning in radiology: an overview of the concepts and a survey of the state of the art with focus on MRI. J Magn Reson Imaging.

[20] Hosny A, Parmar C, Quackenbush J (2018). Artificial intelligence in radiology. Nat Rev Cancer.

[21] Rzecki K, Kucybała I, Gut D (2021). Fully automated algorithm for the detection of bone marrow oedema lesions in patients with axial spondyloarthritis — feasibility study. Biocybern Biomed Eng.

[22] Bhat CS, Chopra M, Andronikou S (2020). Artificial intelligence for interpretation of segments of whole body MRI in CNO: pilot study comparing radiologists versus machine learning algorithm. Pediatr Rheumatol Online J.

[23] Montagnon E, Cerny M, Cadrin-Chenevert A (2020). Deep learning workflow in radiology: a primer. Insights Imaging.

[24] Tardi C (2020) 80–20 rule. Investopedia. https://www.investopedia.com/terms/p/paretoprinciple.asp. Accessed 24 Oct 2021

[25] No authors listed (2021) MedSeg website. https://www.medseg.ai. Accessed 22 Nov 2021

[26] Ronneberger O, Fischer P, Brox T (2015) U-net: convolutional networks for biomedical image segmentation. In: Navab N, Hornegger J, Wells W, Frangi A (eds) Medical image computing and computer-assisted intervention — MICCAI 2015. Springer, Cham

[27] Schneidmuller D, Roder C, Kraus R et al (2011) Development and validation of a paediatric long-bone fracture classification. A prospective multicentre study in 13 European paediatric trauma centres. BMC Musculoskelet Disord 12:89 10.1186/1471-2474-12-89 PMC3096600 21548939

[28] Warfield SK, Zou KH, Wells WM (2004). Simultaneous truth and performance level estimation (STAPLE): an algorithm for the validation of image segmentation. IEEE Trans Med Imaging.

[29] Breivik EK, Björnsson GA, Skovlund E (2000). A comparison of pain rating scales by sampling from clinical trial data. Clin J Pain.

[30] Suther KR, Hopp E, Smevik B (2018). Can visual analogue scale be used in radiologic subjective image quality assessment?. Pediatr Radiol.

[31] Dice RL (1945). Measures of the amount of ecologic association between species. Ecology.

[32] Asgari Taghanaki S, Abhishek K, Cohen JP (2020). Deep semantic segmentation of natural and medical images: a review. Artif Intell Rev.

[33] Bankier AA, Levine D, Halpern EF, Kressel HY (2010). Consensus interpretation in imaging research: is there a better way?. Radiology.

[34] Moore SG, Dawson KL (1990). Red and yellow marrow age-related changes at MR imaging. Radiology.

[35] Zhao Y, Ferguson PJ (2018). Chronic nonbacterial osteomyelitis and chronic recurrent multifocal osteomyelitis in children. Pediatr Clin North Am.

[36] Tang X, Jafargholi Rangraz E, Coudyzer W (2020). Whole liver segmentation based on deep learning and manual adjustment for clinical use in SIRT. Eur J Nucl Med Mol Imaging.

