# Supplementary material for: Automated segmentation of magnetic resonance bone marrow signal: a feasibility study
Source: Pediatr Radiol. 2022 Feb 2;52(6):1104–14. doi: 10.1007/s00247-021-05270-x (PMC9107442; doi:10.1007/s00247-021-05270-x)
Supplement: Supplementary file 1 — Supplementary file1 (DOCX 14 kb) [file 247_2021_5270_MOESM1_ESM.docx]

**Online Supplementary Material 1**

**Training process**
Our selected network architecture was a two-dimensional (2-D) U-Net [26] variant. The model consisted of six down- and up-sampling steps. The model input was three channels (512 × 512 × 3) that corresponded to three consecutive image slices — the slice to be predicted and two neighboring slices. The model output was three channels of the same size (512 × 512 × 3). Each down-sampling step had two 3 × 3 convolutions, a hyperbolic tangent activation function (tanh), followed by 2 × 2 max pooling. Each up-sampling step had 2 × 2 interpolation and two 3 × 3 convolutions with tanh activation. In each step, the convolution output from the down-sampling side was concatenated with the corresponding up-sampling step. The last layer was a 1 × 1 × 3 convolution followed by a sigmoid function. The first and last steps had 16 kernels and the middle of the U-Net (bottleneck layer) had 1,024 kernels, resulting in an 8 × 8 × 1,024 representation. The number of trainable weights in the model was 31,110,035. The highest score for each pixel determined the final segmentation if higher than a threshold of 0.5, with the exception for level-3 signal, where an output of higher than 0.5 always overruled the two other channel outputs.

Of the 85 examinations for training and validation, 15 were manually selected for validation, with a focus on representative bone marrow signal variability. Hyperparameters, augmentation settings and training duration were selected experimentally based on validation data performance. The model was then re-trained including the validation data in training. Test data were never used during the optimisation and training process.

Input images were normalised by subtraction of the mean and division by the standard deviation as computed from the total of the three input images. We used Dice loss function, a batch size of 8, and Adam optimisation with a descending learning rate of 0.0001 down to 0.000001. The model was trained for 500 epochs. During training we applied simple linear augmentations with translation by up to 150 pixels in any direction, up to 25% rotation, 50% scale, 25° shear and left/right flipping in 50% of cases. The programming environment was Python v. 3.6.8, Keras v. 2.2.5 with Tensor flow v. 1.13.1, all open sources. The model was trained using an NVIDIA GTX 1080 Ti graphics processing unit.
